# Mesenchymal Stem Cells Attenuate Diabetic Nephropathy by Suppressing the ERK-Ferroptosis-ROS Axis

**DOI:** 10.3390/ijms27115101

**Published:** 2026-06-04

**Authors:** Shuaijing Ma, Qin Han, Jing Li, Haiyan Wang, Yiming Wang, Xueyuan Bai, Robert Chunhua Zhao

**Affiliations:** 1 Institute of Basic Medical Sciences & School of Basic Medicine, Chinese Academy of Medical Sciences & Peking Union Medical College, Beijing 100005, China; mashuaijing@ibms.pumc.edu.cn (S.M.); hanqin@ibms.pumc.edu.cn (Q.H.); lijing888@ibms.pumc.edu.cn (J.L.); haiyanwork321@163.com (H.W.); wangyiming997@163.com (Y.W.); 2 Beijing Key Laboratory of Artificial Intelligence and Cell-Based Medical Engineering for Interdisciplinary Innovation and Clinical Translation (BZ-2025-119), Beijing 100005, China; 3 State Key Laboratory of Common Mechanism Research for Major Diseases, Beijing 100005, China; 4 Senior Department of Nephrology, Chinese PLA General Hospital, State Key Laboratory of Kidney Diseases, National Clinical Research Center for Kidney Diseases, Beijing 100853, China; 5 Beijing Key Laboratory of Medical Devices and Integrated Traditional Chinese and Western Drug Development for Severe Kidney Diseases, Beijing 100853, China

**Keywords:** mesenchymal stem cells, ferroptosis, oxidative stress, renal tubular injury, MAPK/ERK pathway

## Abstract

Diabetic nephropathy (DN) is a leading cause of end-stage renal disease with limited therapeutic options. Ferroptosis contributes to renal tubular injury in DN. This study investigates whether mesenchymal stem cells (MSCs) ameliorate DN by inhibiting ferroptosis and elucidates the underlying mechanism. In a rat model of type 2 DN, MSCs transplantation improved renal function and histopathology, while reducing mitochondrial dysfunction, iron overload, and ROS-driven ferroptosis. In vitro, MSCs reversed high glucose-induced ferroptosis hallmarks in tubular epithelial cells. Mechanistically, RNA sequencing identified the MAPK/ERK pathway as key. MSCs suppressed the p-ERK/ERK-GPX4/ACSL4 axis, preventing glutathione depletion and lipid peroxidation. Activation of ERK abolished MSCs’ protection, whereas ERK inhibition mimicked it. These findings reveal that targeting ERK-mediated ferroptosis in renal tubules offers a novel therapeutic strategy, with MSCs acting through this specific mechanism.

## 1. Introduction

Diabetic nephropathy (DN) is one of the most serious microvascular complications of diabetes mellitus and is the leading cause of chronic kidney disease and end-stage renal disease globally [1,2,3]. Traditional perspectives considered DN a glomerulocentric disease. However, recent research has revealed that tubular injury may precede glomerulopathy and play a critical role in the early stages of DN [4,5]. The pathogenesis of DN involves multifactorial mechanisms, including dysregulation of lipid metabolism, hemodynamic abnormalities, inflammation, oxidative stress, cellular damage, and ferroptosis [6]. Ferroptosis induces renal tubular epithelial cell death, triggering pathological cascades through damage-associated molecular pattern release, activating innate immunity, disrupting tubular reabsorption, causing proteinuria, and promoting renal fibrosis via epithelial-mesenchymal transition [5,7]. Proximal tubular reabsorption critically demands iron for ATP production [8], and dysregulated iron metabolism secondary to diabetic renal injury heightens oxidative stress and inflammatory responses, thereby potentiating renal damage [9,10,11]. The vicious iron metabolism–oxidative stress cycle constitutes the core mechanism underlying ferroptosis in renal tubular epithelial cells during DN. The above reports suggested the importance of ferroptosis in the pathological progression of diabetic nephropathy.

Mesenchymal stem cells (MSCs) have garnered increasing attention as a novel regenerative therapy for DN. The therapeutic efficacy of MSC transplantation for DN has been established in numerous preclinical studies and demonstrates promising outcomes in early-phase clinical trials [12,13,14,15,16]. However, the clinical translation of these therapies remains challenging, primarily because of heterogeneous therapeutic responses stemming from unresolved mechanistic uncertainties. MSC-based therapies ameliorate diabetic nephropathy primarily via paracrine and immunomodulatory mechanisms, including anti-inflammatory, antioxidant, antifibrotic, and cellular protective effects, alongside promotion of angiogenesis, mitochondrial transfer, and tissue repair [16,17]. Nevertheless, the precise regulatory mechanisms underlying these therapeutic effects remain incompletely elucidated.

Given the established role of ferroptosis in driving renal tubular cell loss and dysfunction in diabetic nephropathy, we hypothesized that the beneficial effects of MSC therapy result from modulation of tubular ferroptosis pathways. Recent studies report that MSCs and their derived exosomes mitigate acute multi-organ injury by regulating ferroptosis [18,19,20,21,22]. However, how MSCs treat chronic metabolic diseases such as DN by modulating ferroptosis remains unclear. To address this gap, we investigated whether human umbilical cord-derived MSCs (UMSCs) ameliorate diabetic kidney injury by targeting the iron metabolism–ROS–ferroptosis positive feedback loop in renal tubular epithelial cells. We evaluated the therapeutic efficacy of UMSCs in DN and explored the underlying molecular mechanisms, with a particular focus on ferroptosis and its upstream signaling pathways.

## 2. Results

### 2.1. UMSCs Mitigate Renal Pathological Manifestations in DN Rat Models

**Figure 1:**
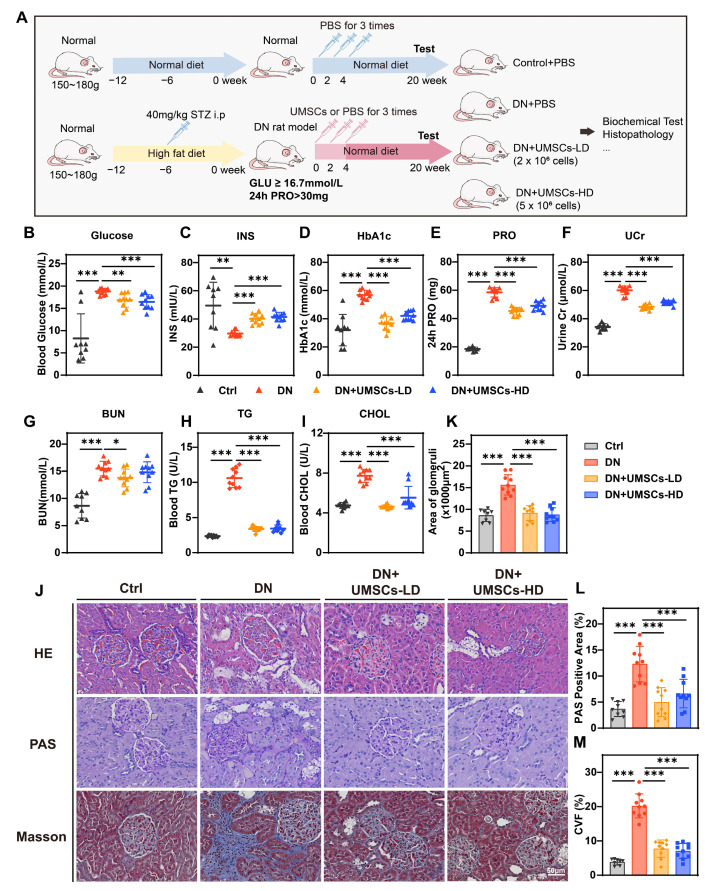
Effect of UMSCs on renal function and morphological features in DN rats. (**A**) Animal experimental design. (**B**–**I**) Changes in blood glucose, insulin—INS, HbA1c, urinary protein—PRO, creatinine—UCr, urea nitrogen—BUN, triglycerides—TG, and total cholesterol—CHOL levels in DN rats treated with or without UMSCs. (**J**) Representative pathological staining of kidney sections (Top panel: HE staining, Middle panel: PAS staining, Bottom panel: Masson staining, Scale bar = 50 μm). (**K**) Quantification analysis of glomerular area. (**L**) Quantitative percentage of PAS-positive area. (**M**) Quantitative analysis of collagen volume fraction, *n* = 9/10; * *p* < 0.05, ** *p* < 0.01, *** *p* < 0.001.

To investigate the therapeutic potential of UMSCs in DN, we established a type 2 DN (T2DN) rat model (GLU ≥ 16.7 mmol/L and 24-h PRO > 30 mg, Supplementary Figure S1) through a combination of a high-fat diet for 6 weeks and intraperitoneal STZ administration. UMSCs were administered via triple tail vein injections (0, 2, and 4 weeks) at two dosage regimens: 2 × 10^6^ cells/injection (Low-dose group, UMSCs-LD, *n* = 10) and 5 × 10^6^ cells/injection (high-dose group, UMSCs-HD, *n* = 10) (Figure 1A and Supplementary Figure S2). Biochemical results detected every four weeks during treatment are presented in Supplementary Figure S3. At the 20-week endpoint, the DN cohort (*n* = 10) exhibited marked elevations in fasting blood glucose, glycated hemoglobin (HbA1c), 24h-urinary protein excretion (PRO), urine creatinine (UCr), and urea nitrogen (BUN), all of which were substantially attenuated by UMSCs therapy (Figure 1B,D–G). However, no clear dose-dependent therapeutic effect of UMSCs was observed for most of the measured parameters. Although serum insulin levels were significantly reduced in the DN group, they returned to normal after UMSCs administration (Figure 1C), indicating that UMSCs ameliorate renal function in diabetic nephropathy. Moreover, the significant reduction in serum lipids (triglycerides and cholesterol) in DN rats following UMSC administration (Figure 1H,I) suggests that lipid lowering is a novel aspect of their pleiotropic effects, beyond their established roles in improving renal function.

Histopathological analysis revealed characteristic renal pathology in DN rats, including glomerular hypertrophy, tubular dilatation, and lipid vacuolization in proximal tubular cells (Figure 1J,K). PAS staining demonstrated tubular basement membrane thickening (Figure 1J,L), while Masson trichrome staining confirmed progressive interstitial fibrosis (Figure 1J,M). Remarkably, UMSCs administration ameliorated these structural anomalies, restoring glomerular morphology and reducing collagen deposition by 40–60% across histological metrics. No persistent human cells were detected in kidney tissues at 20 weeks (Supplementary Figure S4), indicating that therapeutic effects were not mediated by long-term engraftment. These findings confirm the establishing of a successful T2DN model and demonstrate the potent renoprotective efficacy of UMSCs to alleviate both functional and structural renal deterioration.

These findings confirm the establishment of a relevant T2DN model with key metabolic and nephropathic features (Figure 1B–M). UMSCs therapy demonstrated potent nephroprotection by mitigating both functional decline (proteinuria, azotemia) and structural damage (glomerular hypertrophy, fibrosis), highlighting its renoprotective potential in diabetic kidney disease.

### 2.2. UMSCs Attenuate Ferroptosis-Related Mitochondrial Dysfunction in DN Renal Tissues

**Figure 2:**
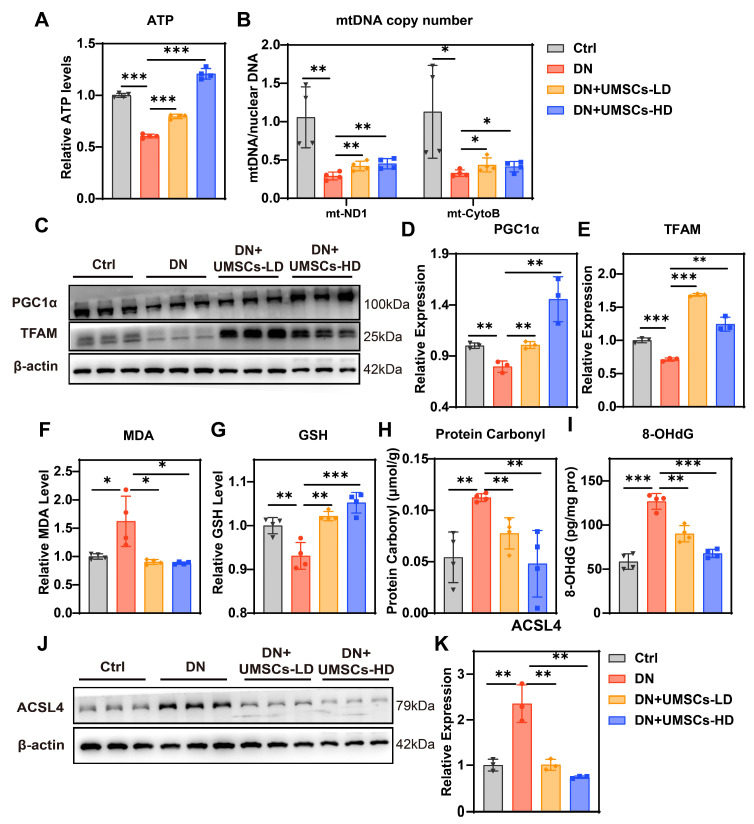
UMSCs alleviate renal ferroptosis-related mitochondrial dysfunction in DN rats. (**A**) ATP levels in rat renal tissues, *n* = 4. (**B**) MtDNA copy number in rat renal tissues, *n* = 4. (**C**–**E**) Protein expression of PGC1α and TFAM detected by western blot and quantitative analysis in rat renal tissues, *n* = 3. (**F**–**I**) MDA, GSH, PCO, and 8-OHdG levels in rat renal tissues, *n* = 4. (**J**,**K**). Protein expression of ACSL4 detected by Western blot in rat renal tissues and quantitative analysis, *n* = 3; * *p* < 0.05, ** *p* < 0.01, *** *p* < 0.001.

Ferroptosis is characterized by complex and dynamic mitochondrial damage. To investigate the role of mitochondria in the pathogenesis of DN, we first assessed mitochondrial function in the renal tissues of DN rats. Results demonstrated significantly reduced ATP production in renal tissues from DN rats, accompanied by decreased mitochondrial DNA (mtDNA) copy number (Figure 2A,B). UMSCs treatment significantly restored mitochondrial function. Western blot analysis revealed suppressed expression of mitochondrial biogenesis markers PGC1α and TFAM in DN kidneys, which was reversed by UMSCs (Figure 2C–E), indicating that impaired mitochondrial biogenesis critically contributes to mitochondrial dysfunction during severe glucose-induced renal damage and that UMSCs may confer protection by enhancing mitochondrial biogenesis and promoting functional recovery.

Mitochondria constitute the primary cellular source of ROS [23]. Exposure to metabolic stressors (e.g., hyperglycemia and inflammation) induces mitochondrial damage, triggering excessive ROS generation [24] that promotes lipid peroxidation and accelerates ferroptosis. Renal oxidative stress markers were assessed in DN rats. Compared with controls, DN rats exhibited significantly elevated levels of malondialdehyde (MDA), a terminal lipid peroxidation product, along with depleted glutathione (GSH). UMSCs treatment attenuated MDA accumulation and restored GSH levels (Figure 2F,G). Protein carbonyl content (PCO) and 8-hydroxy-2′-deoxyguanosine (8-OHdG) were also increased in DN kidneys and reduced by UMSCs, indicating mitigation of protein and DNA oxidation (Figure 2H,I). Additionally, ACSL4, a pro-ferroptosis enzyme, was upregulated in DN and attenuated by UMSCs (Figure 2J,K). Collectively, these results demonstrate robust oxidative stress and lipid peroxidation in DN kidneys, supporting the involvement of ferroptosis in DN pathogenesis.

### 2.3. UMSCs Attenuate Ferroptosis in DN Rat Renal Tissues

**Figure 3:**
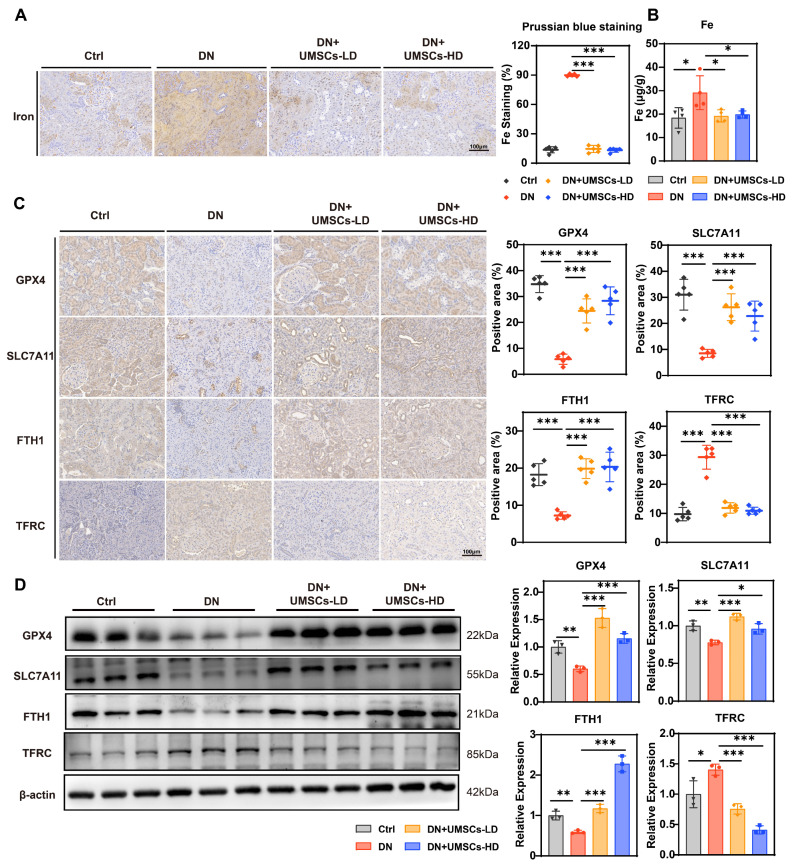
UMSCs inhibit ferroptosis in rat renal tissues with DN. (**A**) Rat renal tissues with Prussian blue staining (DAB-enhanced method) and statistical analysis (Scale bar = 100 μm), *n* = 5. (**B**) Fe levels in rat renal tissues, *n* = 4. (**C**) Immunohistochemistry staining for GPX4, SLC7A11, FTH1, and TFRC in rat renal tissues and quantitative analysis of positive areas (Scale bar = 100 μm), *n* = 5. (**D**) Protein expression of ferroptosis markers GPX4, SLC7A11, FTH1, and TFRC in rat renal tissues and quantitative analysis of relative protein expression, *n* = 3; * *p* < 0.05, ** *p* < 0.01, *** *p* < 0.001.

To investigate the potential mitigating effects of UMSCs on ferroptosis in DN, we initially examined renal iron deposition, a hallmark of ferroptosis. Prussian blue staining revealed marked iron accumulation in DN rat kidneys, which was attenuated by UMSCs (Figure 3A). Quantitative iron analysis confirmed renal iron overload and disrupted iron homeostasis in diabetic kidneys, with prominent deposition in tubular epithelial cells (Figure 3A,B).

To further elucidate the involvement of ferroptosis and the regulatory role of UMSCs, we then examined expression of key ferroptosis regulators. Immunohistochemistry showed downregulation of the ferroptosis inhibitors GPX4, SLC7A11, and FTH1, alongside upregulation of the iron transporter TFRC in DN renal tissues, particularly in tubules, indicating tubular susceptibility to ferroptosis. UMSCs treatment restored these expression profiles toward normal (Figure 3C). Western blot analysis corroborated these protein expression changes in DN kidneys and confirmed that UMSCs intervention reversed the aberrant patterns (Figure 3D). These findings demonstrate that ferroptosis contributes to DN pathogenesis and that UMSCs alleviate renal injury by modulating this pathway.

### 2.4. UMSCs-CM Mitigates High Glucose-Induced Ferroptosis in HK-2 Cells

**Figure 4:**
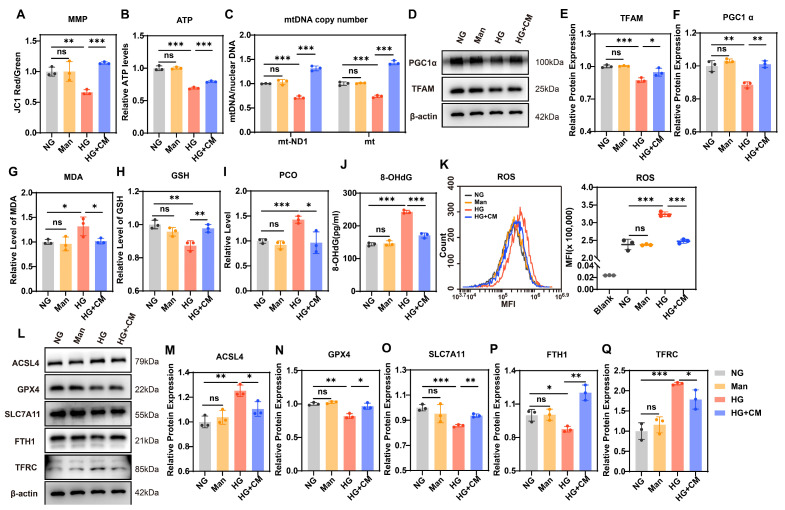
UMSCs-CM reduces high glucose-induced ferroptosis in HK-2 cells. (**A**) HK-2 cells mitochondrial membrane potential detected by JC-1 probe, *n* = 3. (**B**) Total ATP level in HK-2 cells, *n* = 3. (**C**) mtDNA copy number in HK-2 cells, *n* = 3; (**D**–**F**) Protein expression of PGC1α and TFAM in HK-2 cells and quantitative analysis, *n* = 3. (**G**–**I**) MDA, GSH, and PCO levels in HK-2 cells, *n* = 3. (**J**) Supernatant 8-OHdG level in HK-2 cells, *n* = 3. (**K**) Flow assay for ROS level in HK-2 cells. (**L**–**Q**) Protein expression of ferroptosis markers ACSL4, GPX4, SLC7A11, FTH1, and TFRC in HK-2 cells and quantitative analysis, *n* = 3; * *p* < 0.05, ** *p* < 0.01, *** *p* < 0.001 Blank: No DCFH-DA incubation; NG—normal glucose; Man—mannitol, osmotic control; HG—high glucose; HG+CM—High glucose plus UMSCs-CM.

Tubular injury constitutes a critical determinant of DN progression [10,11,25]. To investigate the underlying mechanisms, we evaluated the effects of high glucose on ferroptosis in human renal tubular epithelial cells (HK-2). Following exposure to high glucose (30 mM; HG) for 72 h, HK-2 cells exhibited ferroptosis-associated mitochondrial dysfunction, as evidenced by reduced mitochondrial membrane potential (MMP, ΔΨm, Figure 4A and Supplementary Figure S5A), decreased ATP production (Figure 4B), and lower mtDNA copy number (Figure 4C). Treatment with UMSCs-conditioned medium (UMSCs-CM; HG+CM) restored these parameters (Figure 4A–C), indicating improved mitochondrial function under glucose stress. Western blotting showed downregulation of PGC1α and TFAM in HK-2 cells under high glucose conditions, which was reversed by UMSCs-CM (Figure 4D–F and Supplementary Figure S5B). These findings suggest that the therapeutic benefit of UMSCs-CM is mediated by enhancing of mitochondrial biogenesis and the attenuation of severe glucose-induced mitochondrial damage.

Given the link between ferroptosis and mitochondrial dysfunction in HG-induced injury, we next assessed oxidative stress in vitro. High-glucose conditions induced redox imbalance in HK-2 cells, as evidenced by increased MDA and reduced GSH levels. UMSCs-CM treatment attenuated MDA accumulation and normalized GSH (Figure 4G,H). PCO content was elevated in HG-treated cells and reduced by UMSCs-CM (Figure 4I). Similarly, 8-OHdG levels increased in culture supernatants and were lowered by UMSCs-CM (Figure 4J). Intracellular ROS accumulation, detected by flow cytometry, was also mitigated by UMSCs-CM (Figure 4K and Supplementary Figure S6B). Together, these results demonstrate that UMSCs-CM alleviates HG-induced oxidative stress by reducing lipid peroxidation, protein carbonylation, DNA oxidation, and overall ROS levels.

Western blot analysis of ferroptosis markers revealed that HG exposure upregulated ACSL4 and TFRC expression and downregulated GPX4, SLC7A11, and FTH1 expression in HK-2 cells (Figure 4L–Q and Supplementary Figure S6C), consistent with ferroptosis induction. UMSCs-CM treatment reversed these expression changes, indicating attenuation of HG-induced ferroptosis in renal tubular epithelial cells.

### 2.5. UMSCs Exert Therapeutic Effects via Suppression of the MAPK/ERK Pathway in DN Renal Tissues

**Figure 5:**
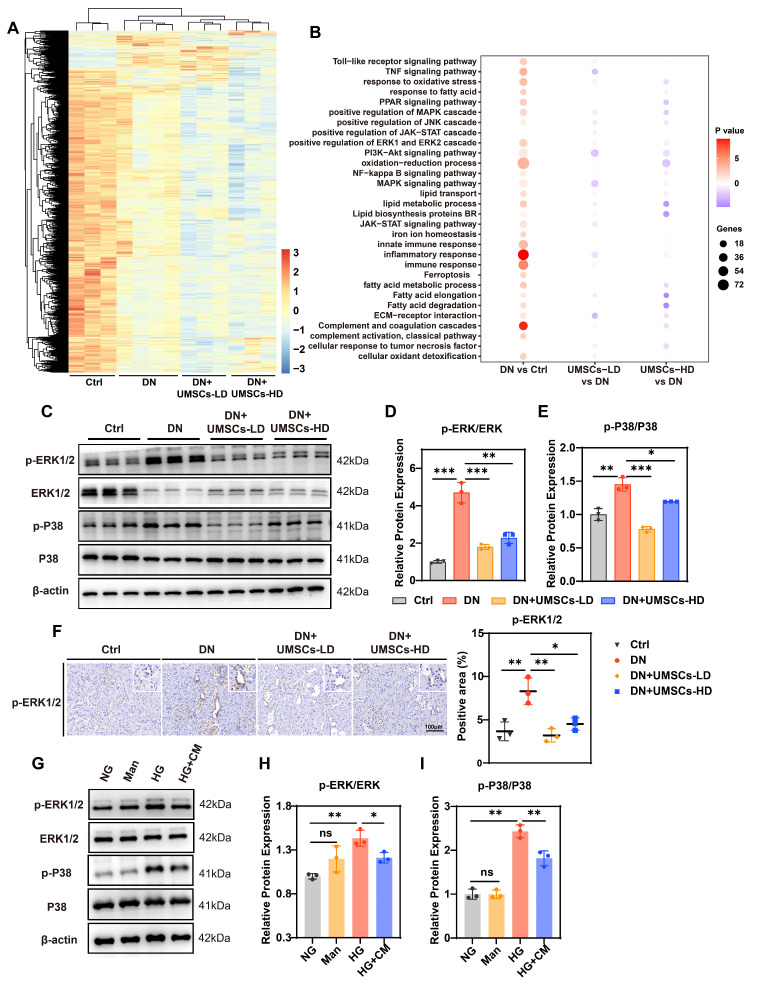
UMSCs exert therapeutic effects by inhibiting p-ERK/ERK activation in renal tissues with DN. (**A**) GSEA analysis. (**B**) GO analysis. (**C**–**E**) Western blot detection of p-ERK/ERK and p-P38/P38 expression in rat kidney tissues, and quantitative analysis of the ratios, *n* = 3. (**F**) Immunohistochemistry detection of p-ERK expression in rat kidney tissues and quantitative analysis (Scale bar = 100 μm), *n* = 3. (**G**–**I**) Western blot detection of p-ERK/ERK, p-P38/P38 expression and quantitative analysis of ratio in HK-2 cells, *n* = 3; * *p* < 0.05, ** *p* < 0.01, *** *p* < 0.001. NG—normal glucose; Man—mannitol, osmotic control; HG—high glucose; HG+CM—high glucose plus UMSCs-CM.

To investigate the mechanisms by which UMSCs inhibit ferroptosis and protect against DN, we performed bulk RNA sequencing on renal tissues. Gene set enrichment analysis (GSEA) and GO analysis revealed that genes associated with ferroptosis, iron ion homeostasis, and lipid metabolism were significantly upregulated in DN renal tissues (Figure 5A,B). UMSC administration markedly reversed these transcriptional changes, consistent with observed phenotypic improvements.

Notably, transcriptomic profiling revealed activation of the MAPK/ERK pathway in DN, evidenced by an elevated p-ERK/ERK ratio, which was attenuated following UMSC administration (Figure 5B). As a key MAPK family member, ERK responds to growth factors, cytokines, and stress stimuli, regulating proliferation, differentiation, survival, and metabolism [26]. Given the role of ERK in cellular stress responses, we hypothesized that the therapeutic effect of UMSCs is mediated by inhibiting p-ERK/ERK activation. Western blot confirmed increased phosphorylation of ERK and p38 in DN kidneys, which was normalized by UMSCs treatment (Figure 5C–E). Immunohistochemistry further validated these findings, showing reduced p-ERK staining in UMSCs-treated renal sections (Figure 5F).

To validate the translational relevance of these findings, we established an in vitro model using HK-2 cells cultured in high glucose. Consistent with the in vivo data, high glucose induced the phosphorylation of ERK and P38, which was attenuated by UMSCs-CM (Figure 5G–I). These results demonstrated that UMSCs suppressed aberrant MAPK/ERK activation in diabetic kidneys. Given the established link between ERK signaling, ferroptosis, and renal injury, our findings suggest that UMSCs attenuate ferroptosis and delay DN progression primarily by inhibiting the MAPK/ERK pathway.

### 2.6. UMSCs Attenuate Ferroptosis-ROS Loop via p-ERK/ERK Pathway Suppression in HK-2 Cells

**Figure 6:**
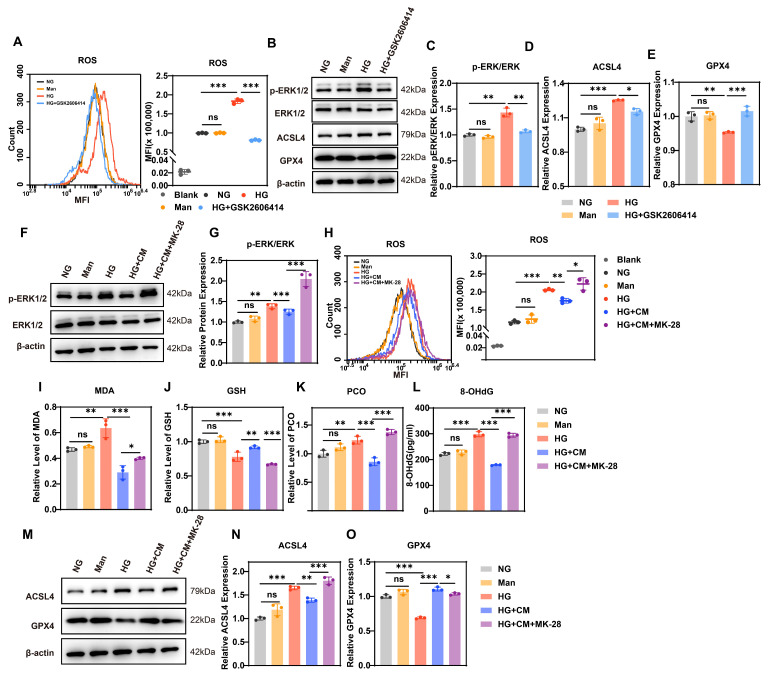
UMSCs inhibit ferroptosis-ROS loop by suppressing p-ERK/ERK activation in HK-2 cells (**A**) Flow assay of ROS levels in HK-2 cells after application of p-ERK inhibitor GSK2606414 (10 nM) in HG group, *n* = 3. (**B**–**E**) Western blot analysis showing the expression change of p-ERK/ERK, ACSL4, GPX4 in HK-2 cells treated with p-ERK inhibitor GSK2606414 (10 nM) and quantitative analysis, *n* = 3. (**F**,**G**) Western blot detection of p-ERK/ERK expression in HK-2 cells after application of p-ERK activator MK-28 (10 μM) in the UMSCs CM-treated group and quantitative analysis of p-ERK/ERK ratio, *n* = 3. (**H**) Flow assay for ROS level in HK-2 cells, *n* = 3. (**I**) MDA level in HK-2 cells, *n* = 3. (**J**) GSH level in HK-2 cells, *n* = 3. (**K**) PCO level in HK-2 cells, *n* = 3. (**L**) 8-OHdG level in HK-2 cell supernatant, *n* = 3. (**M**–**O**). Western blot detection of ACSL4, GPX4 protein expression in HK-2 cells and quantitative analysis, *n* = 3. * *p* < 0.05, ** *p* < 0.01, *** *p* < 0.001 Blank—No DCFH-DA incubation. NG—Normal glucose. Man—Mannitol, osmotic control. HG—High glucose. HG+ GSK2606414—High glucose plus p-ERK inhibitor GSK2606414 (10 nM). HG+CM—High glucose plus UMSCs-CM. HG+CM+MK-28—High glucose plus UMSCs-CM and p-ERK activator MK-28 (10 μM).

To pharmacologically validate the role of ERK activation in high-glucose-induced renal tubular ferroptosis, we utilized the selective ERK inhibitor GSK2606414 at a concentration of 10 nM. As anticipated, treatment with GSK2606414 markedly attenuated HG-stimulated ROS overproduction in HK-2 cells (Figure 6A,B). Concurrently, this inhibition of ERK signaling significantly suppressed the expression of the pro-ferroptosis protein ACSL4, while upregulating the key ferroptosis inhibitor GPX4 (Figure 6B–E). These data collectively confirm that pharmacological blockade of p-ERK effectively mitigates high glucose-induced ferroptosis in renal tubular cells.

To further test pathway specificity in UMSCs-mediated protection, the ERK activator MK-28 (10 μM) was applied to UMSCs-CM-treated cells. MK-28 treatment effectively induced ERK phosphorylation (Figure 6F,G) and partially reversed the renoprotective effects of UMSCs-CM. Specifically, MK-28 co-treatment abolished the UMSCs-CM-mediated reductions in oxidative stress markers, including MDA, PCO, 8-OHdG, and intracellular ROS levels (Figure 6H–L). Furthermore, it diminished the restoration of GSH levels, indicating a compromised antioxidant capacity. In parallel, the activation of ERK attenuated the ferroptosis protection conferred by UMSCs-CM, as evidenced by a decrease in GPX4 expression and a concomitant increase in ACSL4 levels (Figure 6M–O).

**Figure 7:**
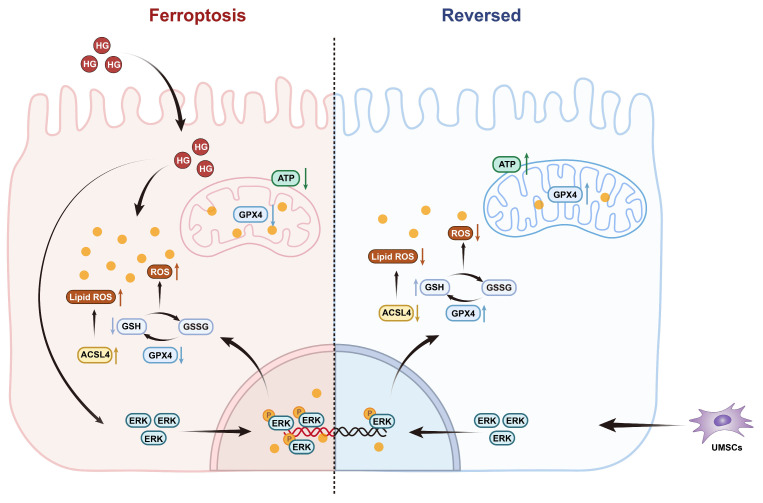
Molecular mechanism of UMSCs in the treatment of diabetic nephropathy.

Collectively, using both inhibitor and activator approaches, we demonstrate that UMSC-CM protects against HG-induced tubular ferroptosis by suppressing the p-ERK/ERK pathway. This suppression alleviates downstream oxidative stress, as reflected by reduced ROS, MDA, PCO, and 8-OHdG, and restores antioxidant capacity by enhancing GSH levels. Mechanistically, p-ERK inhibition downregulates ACSL4 and upregulates GPX4, thereby blocking ferroptosis. These findings are schematically illustrated in Figure 7, which depicts the HG-triggered ERK activation cascade leading to ferroptosis and the protective intervention by UMSCs.

## 3. Discussion

In this study, we demonstrate the therapeutic efficacy of UMSCs in DN rats and elucidate the underlying molecular mechanisms. Under hyperglycemic conditions, pathological activation of the p-ERK/ERK signaling pathway in renal tubular epithelial cells drives ferroptosis by suppressing GPX4 and excessive ROS production, which results in oxidative damage to DNA, proteins, and lipids. UMSCs mitigate renal injury by inhibiting hyperglycemia-induced ERK phosphorylation, which in turn suppresses the ferroptosis-ROS feedback loop and attenuates oxidative stress (Figure 7).

Consistent with prior reports [13,14,15], UMSCs demonstrated renoprotective effects in high-fat diet/STZ-induced T2DN rats. Both low (2 × 10^6^) and high-dose (5 × 10^6^) groups showed reduced blood glucose, elevated insulin, and improved renal function, though without dose dependence. This absence of dose-dependence aligns with findings in T1DN mice [27] and a phase 1 systemic lupus erythematosus trial (2 × 10^6^ to 4 × 10^6^ cells/kg) [28], whereas some spinal cord injury models report dose-dependent MSC effects (4 × 10^5^ to 10^6^ cells) [29], potentially due to lower doses used. Our lack of dose dependence may reflect supra-threshold dosing in this model. Pathological assessment confirmed early-stage DN, lacking advanced features like Kimmelstiel–Wilson nodules, consistent with evidence that tubular injury precedes glomerular damage in DN progression [4,30]. Notably, UMSCs significantly reduced serum triglycerides and total cholesterol, along with glucose, suggesting multifaceted metabolic benefits and potential utility in lipid management.

Our results demonstrate that DN rats exhibit renal tubular epithelial cell iron overload, mitochondrial dysfunction, and excessive oxidation affecting DNA, proteins, and lipids, culminating in ferroptosis. UMSCs ameliorated these abnormalities, indicating multi-target effects. Ferroptosis is an iron-dependent form of regulated cell death driven by lipid peroxidation [31], with mitochondrial damage acting as both trigger and amplifier [32,33]. In chronic hyperglycemia, mitochondrial injury promotes ROS overproduction, which, together with iron accumulation, PUFA-rich membranes, and GPX4 inactivation, facilitates mitochondrial ferroptosis [34,35,36]. UMSCs disrupted this “mitochondria-iron-ROS-ferroptosis” cascade, preserving mitochondrial integrity and limiting oxidative damage.

We identify a novel role for the MAPK/ERK pathway in mediating ferroptosis in DN and its modulation by UMSCs. Ferroptosis is regulated via AMPK, Akt/mTOR, MAPK, and p53/NRF2/ATF4 axes [9,32,36,37,38,39]. While MAPK typically promotes ferroptosis through JNK/p38-mediated ACSL4/LOX activation [9,32,38], ERK has been shown to promote ferroptosis in some cancers by inhibiting System Xc^−^ [39]. In this study, RNA-seq analysis revealed that UMSCs suppress ferroptosis by inhibiting the MAPK/ERK pathway. Pharmacological intervention in HG-injured HK-2 cells using the ERK phosphorylation inhibitor GSK2606414 or the ERK activator MK-28 demonstrated that ERK inhibition effectively attenuated HG-induced ROS overproduction and ferroptosis. Conversely, ERK activation diminished the therapeutic efficacy of UMSCs against HG-induced oxidative stress and ferroptosis. These findings support ERK inhibition as a therapeutic strategy in DN. Although ERK inhibitors are extensively investigated in oncology, clinical translation is limited by feedback activation, toxicity, and resistance [40]. Combining ERK inhibitors with UMSCs may offer a novel therapeutic approach. Notably, hyperglycemia-activated p-ERK may enhance HIF 1α stability via ROS-mediated EGLN inactivation or direct phosphorylation, thereby upregulating iron uptake and exacerbating mitochondrial dysfunction [41,42]. We hypothesize that UMSCs disrupt this ERK-HIF 1α-ferroptosis positive feedback loop by inhibiting ERK phosphorylation. Although HIF-1α/EGLNs were not directly examined here, they warrant further investigation as downstream effectors of the anti-ferroptotic effects of UMSCs.

Notably, our results demonstrated that no human cells were detected in kidney tissues at 20 weeks after MSCs transplantation (Supplementary Figure S4), suggesting that therapeutic effects are mediated primarily via paracrine mechanisms rather than direct engraftment or differentiation [43]. This supports the emerging paradigm that MSCs exert sustained benefits via transient secretion of factors (e.g., exosomes, cytokines) that modulate the host microenvironment. Several limitations should be acknowledged. First, the specific secretome components responsible for ERK inhibition remain unidentified and warrant further investigation. Second, while this study focused on ERK-mediated ferroptosis, other pathways implicated in DN (e.g., TGF-β/Smad, NF-κB) may also be modulated by UMSCs [44] and merit future exploration.

## 4. Materials and Methods

### 4.1. Culture and Conditioned Medium Collection from UMSCs

The primary human umbilical cord-derived MSCs (UMSCs) were provided by Cell Energy Life Sciences Group Co. Ltd., Qingdao, China (Initial ethical approval by the Ethics Committee of Liaocheng People’s Hospital, Approval No. 2021105, and the donors had signed informed consent). UMSCs were identified as described previously [45] (Supplementary Figure S7). UMSCs of passage 4 or 5 were used in our experiments. Cells were cultured in a humidified incubator with 5% CO_2_ at 37 °C and passaged with trypsin/EDTA after reaching confluence.

Once the UMSCs reached 70–80% confluency, the medium was replaced with fresh full medium, and the cells were harvested after 24 h. Subsequently, UMSCs-CM were centrifuged at 3000 rpm for 20 min, followed by filtration through a 0.22 μm filter to remove detached MSCs and cell debris.

### 4.2. Animal Model and Treatment Protocols

Sprague-Dawley (SD) rats were provided by Spefo (Beijing) Biotechnology Co., Ltd. (Beijing, China), and all animal procedures followed guidelines approved by the Experimental Animal Ethics Committee of Kangtai Medical Laboratory Service Hebei Co., Ltd., Langfang, China (Approval No. MDL2021-11-18-02). After acclimation, male SD rats (5–6 weeks old, 150–180 g) were fed a high-fat diet for 6 weeks to induce insulin resistance, followed by a 12–18 h fast and a single intraperitoneal injection of streptozotocin (STZ, 40 mg/kg). Blood glucose was monitored daily for three days. Rats with sustained glucose ≥ 16.7 mmol/L were considered type 2 diabetes (Supplementary Figure S1A). Type 2 diabetic nephropathy (T2DN) was confirmed when the 24-h urinary protein (PRO) excretion exceeded 30 mg (Supplementary Figure S1B). On day 0, 35 rats meeting both criteria (GLU ≥ 16.7 mmol/L and 24-h PRO > 30 mg) were selected as established T2DN models (Supplementary Figure S2).

Subsequently, thirty of these T2DN rats were randomly selected and divided into 3 groups (Supplementary Figure S2, *n* = 10 per group): the DN model group (DN), which received no cell therapy; the low-dose umbilical cord mesenchymal stem cell group (UMSCs-LD), administered 2 × 10^6^ cells per rat; and the high-dose group (UMSCs-HD), administered 5 × 10^6^ cells per rat. Cell suspensions were delivered via intravenous injection every 2 weeks for a total of 3 injections. Meanwhile, both the DN model group and a normal control group of SD rats (Ctrl, *n* = 9) received equal volumes of PBS via the same route and schedule. The timelines for model induction and treatment administration are presented in Figure 1A. In vivo, therapeutic efficacy was assessed by renal function parameters (glucose, HbA1c, insulin, creatinine, BUN, and 24-h urinary protein) and renal histopathological staining (HE, PAS, and Masson).

### 4.3. Biochemical Test

The 24 h urine and blood samples were collected from rats at 4-week intervals up to week 20, and analyzed for urea nitrogen (BUN), 24 h urinary protein (PRO), creatinine (Cr), blood glucose, insulin, glycosylated hemoglobin (HbA1c), triglycerides (TG), and total cholesterol (CHOL) levels according to the manufacturer’s instructions.

### 4.4. Pathological Staining

Renal tissues were fixed in 4% paraformaldehyde, paraffin-embedded, and sectioned at 5 μm. After hydration through graded ethanol (100%, 95%, 70%, and 30%) and distilled water (2 min each), sections were subjected to hematoxylin and eosin (HE), periodic acid–Schiff (PAS), and Masson’s trichrome staining to assess glomerular structure, mesangial matrix expansion, basement membrane changes, and interstitial fibrosis. All stained sections were evaluated by a blinded pathologist. For each slide, three random fields were quantitatively analyzed using ImageJ v1.54g.

### 4.5. Immunohistochemistry

Kidney sections were deparaffinized, rehydrated, and subjected to antigen retrieval in 10 mM sodium citrate buffer for 20 min. After blocking with 5% BSA for 1 h at room temperature, slides were incubated overnight at 4 °C with primary antibodies (see Supplementary Methods). Following incubation with secondary antibodies, signals were developed using DAB and counterstained with hematoxylin. Staining intensity was semi-quantitatively analyzed using ImageJ software in at least three random 20× fields per section selected by an independent pathologist (*n* = 5).

### 4.6. Cell Culture and Intervention

The human renal tubular epithelial cell line HK-2 (CCTCC No. GDC0152, Wuhan, China) was cultured in MEM (C11095500BT, Gibco, Waltham, MA, USA) and divided into four groups: normal glucose (5 mM, NG), high glucose (30 mM, HG), HG plus UMSCs-CM (HG+CM), and osmotic control (5 mM glucose + 24.5 mM mannitol, Man). To investigate the role of the p-ERK pathway, HG-cultured cells were treated with 10 nM GSK2606414 (HY-18072, MCE, Princeton, NJ, USA) for 72 h. To determine whether UMSCs-CM inhibits ferroptosis via p-ERK/ERK, HG cells were co-treated with UMSCs-CM and 10 μM MK-28 (HY-137207, MCE) for 72 h. In vitro, efficacy was evaluated in HK-2 cells under high glucose by measuring oxidative stress markers (ROS, MDA, GSH, PCO, and 8-OHdG) and mitochondrial function (mitochondrial membrane potential, ATP levels); MAPK/ERK involvement was confirmed using pathway inhibitors/activators with total ROS as the readout.

### 4.7. ELISA Detection of 8-OHdG

The levels of 8-OHdG in kidney tissues and HK-2 cells were determined using ELISA kits (MM-0331H1, Meimian, Yancheng, China) according to the manufacturer’s instructions.

### 4.8. Malondialdehyde (MDA) Assay

According to the MDA Assay Kit (S0131S, Beyotime, Shanghai, China), MDA levels in kidney tissues or HK-2 cells were measured after lysis and incubation using a microplate reader.

### 4.9. Glutathione (GSH) Assay

Total GSH content in tissues or HK-2 cells was assessed using the Reduced Glutathione Content Assay Kit (BC1175, Solarbio, Beijing, China) following the manufacturer’s instructions.

### 4.10. Protein Carbonyl Content (PCO) Test

The levels of PCO in kidney tissues and HK-2 cells were determined by Protein Carbonyl Content Assay Kit (BC1275, Solarbio) following the manufacturer’s instructions.

### 4.11. Prussian Blue Staining (Enhance with DAB)

Kidney sections were deparaffinized, rehydrated, and stained with Prussian blue, followed by DAB incubation for 2 min. After hematoxylin counterstaining, three random non-overlapping fields per slide were selected by a blinded pathologist and quantified using ImageJ (*n* = 5).

### 4.12. Iron Array

Total iron levels in tissues were assessed using a tissue iron assay kit (BC4355, Solarbio, China) following the manufacturer’s instructions.

### 4.13. Western Blotting

Western blotting was performed as previously described [46]. Primary antibodies (See Supplementary Methods) were incubated overnight at 4 °C. Secondary antibodies were incubated for 1 h at room temperature. Chemiluminescent detection was performed to visualize the protein bands. Band intensities were quantified using ImageJ software (NIH, Bethesda, MD, USA). The relative changes in protein expression in treated groups compared to control cells or tissues were statistically analyzed.

### 4.14. ATP Level

Following the procedure of the ATP Assay Kit (S0026, Beyotime), the ATP level of kidney tissues and HK-2 cells was detected by chemiluminescence.

### 4.15. Mitochondrial Membrane Potential (MMP) Assay

HK-2 cells were harvested and incubated with the JC-1 probe according to the manufacturer’s protocol for the mitochondrial membrane potential assay kit (M8650, Solarbio). Subsequently, mitochondrial membrane potential was assessed via flow cytometry.

### 4.16. MtDNA Copy Number

Genomic DNA was isolated from kidney tissues or total cells pursuant to the instructions of the DNA extraction kit (DP304, TianGen, Beijing, China). Subsequently, cycle threshold (Ct) values were acquired via quantitative real-time PCR (qPCR) performed with a standard system and procedure. Primer sequences are available in the Supplementary Methods.

### 4.17. ROS Level

HK-2 cells were loaded with DCFH probe (S0033S, Beyotime) in situ according to the instructions and then incubated at 37 °C for 15 min for staining, and photographed by laser confocal microscopy under microscopic observation or assessed via flow cytometry.

### 4.18. RNA-Seq and Analysis

Total RNA was isolated from rat kidney tissues using Trizol (Invitrogen, Carlsbad, CA, USA) and RNeasy Mini Kit (Qiagen, Valencia, CA, USA). rRNA-depleted libraries were prepared with NEBNext Directional RNA Library Prep Kit (NEB, Ipswich, MA, USA) and sequenced on Illumina NovaSeq (Illumina, San Diego, CA, USA). Gene expression was quantified as FPKM, and differential expression analysis was performed using DESeq2 R package (1.30.0) with FDR correction.

### 4.19. Statistical Analysis

All results were analyzed using GraphPad Prism software (version 10.1.2) and expressed as means ± standard deviation (Mean ± SD). Statistical analysis was performed as indicated in the figure legends. For the two-group comparison, a Student’s *t*-test was performed. For the multiple-group comparison, a one-way ANOVA was performed.

## 5. Conclusions

In conclusion, this study elucidates the molecular mechanisms underlying UMSC efficacy, moving beyond descriptive accounts. We demonstrate that UMSCs protect renal parenchymal cells by inhibiting MAPK/ERK phosphorylation, which disrupts the ferroptosis-ROS feedback loop. This mechanistic insight not only validates UMSC-based therapy for DN but also highlights the ERK/ferroptosis axis as a promising target. Targeting this pathway, whether through cell therapy or paracrine mediators, thus holds substantial translational potential.

## Data Availability

The original contributions presented in this study are included in the article/Supplementary Materials. Further inquiries can be directed to the corresponding authors.

## Supplementary Materials

The following supporting information can be downloaded at: https://www.mdpi.com/article/10.3390/ijms27115101/s1.

## Abbreviations

The following abbreviations are used in this manuscript:

MAPK	Mitogen-activated protein kinase
ERK	Extracellular signal-regulated kinase
DN	Diabetic nephropathy
MSCs	Mesenchymal stem cells
UMSCs	Human umbilical cord-derived MSCs
UMSCs-CM	UMSCs-conditioned medium
MDA	Malondialdehyde
GSH	Glutathione
8-OHdG	8-Hydroxy-2′-deoxyguanosine
PCO	Protein carbonyl
ROS	Reactive oxygen species
PRO	24 h urinary protein excretion
MMP	Mitochondrial membrane potential
ATP	Adenosine Triphosphate
