# Supplementary material for: Mesenchymal Stem Cells Attenuate Diabetic Nephropathy by Suppressing the ERK-Ferroptosis-ROS Axis
Source: Int J Mol Sci. 2026 Jun 4;27(11):5101. doi: 10.3390/ijms27115101 (PMC13256801; doi:10.3390/ijms27115101)
Supplement: Supplementary file 1 [file ijms-27-05101-s001.zip › ijms-4247766-supplementary.pdf]

Supplementary Materials and Methods

Culture of UMSCs

The human umbilical cord-derived MSCs (UMSCs) were obtained from Cell Energy Life Sciences group CO. LTD (Initial ethical approval by Ethics Committee of Liaocheng People's Hospital, Approval No. 2021105, and the donors had signed informed consent.). UMSCs were cultured on T75 flasks (Corning, USA) in DMEM/F12 medium (Gibco, USA) with 2% FBS (Gibco, USA), 10 ng/ml platelet-derived growth factor bb (Sigma-Aldrich), 10 ng/ml epidermal growth factor (Sigma-Aldrich), 10^{-4} Mascorbic acid 2-phosphate (Sigma-Aldrich), 10^{-8} M dexamethasone (Sigma-Aldrich), 10^{-9} M insulin transferrin selenium (Gibco), 100 U/ml penicillin, and 100 μg/ml streptomycin (Gibco). The cells were cultured in a humidified incubator at 37°C with 5% CO_2 and passaged with trypsin/EDTA after reaching the confluence.

We detected surface markers on UMSCs via flow cytometry, which revealed that the cells were positive for CD29 (555443, BD Biosciences, USA), CD44 (555479, BD Biosciences, USA), CD73 (550257, BD Biosciences, USA), CD90 (12-0909-42, Invitrogen, USA), and CD105 (12-1057-42, Invitrogen, USA), but negative for CD31 (555446, BD Biosciences, USA), CD34 (550761, BD Biosciences, USA), CD45 (555483, BD Biosciences, USA), CD206 (551135, BD Biosciences, USA), and HLA-DR (555812, BD Biosciences, USA) (Supplementary Fig. S7A). Additionally, we assessed the adipogenic and osteogenic differentiation potentials of the UMSCs (Supplementary Fig. S7B), which confirmed that the cultured cells were MSCs.

Preparation of UMSCs conditioned medium (UMSCs-CM)

Once the UMSCs reached 70-80% confluency, the medium was replaced with fresh full medium and harvested after 24h. Subsequently, UMSCs-CM were centrifuged at 3000 rpm for 20 min with 0.22μm filtration to remove detached MSCs and cell debris.

Animal model and treatment protocols

Sprague-Dawley (SD) rats were supplied by Spefo (Beijing) Biotechnology Co., Ltd. All animal experimental procedures were conducted in accordance with the guidelines approved by the Experimental Animal Ethics Committee of Kangtai Medical Laboratory Service Hebei Co., Ltd. (Approval No. MDL2021-11-18-02). The rats were maintained under specific pathogen-free (SPF) conditions in an environmentally controlled barrier facility, with ad libitum access to food and water, and all efforts were made to minimize animal discomfort.

After acclimation, male SD rats (5–6 weeks old, 150–180 g) were fed a high-fat diet for 6 weeks to induce insulin resistance. Following this dietary induction, the animals were fasted for 12–18 hours with free access to water, and then received a single intraperitoneal injection of streptozotocin (STZ) at a dose of 40 mg/kg. Blood glucose (GLU) levels were monitored daily over the next three consecutive days. Rats that displayed sustained hyperglycemia, defined as a blood glucose level ≥ 16.7 mmol/L, were considered to have developed type 2 diabetes (Supplementary Fig. S1A). To confirm the development of type 2 diabetic nephropathy (T2DN), 24-hour urinary protein (PRO) excretion was measured every four weeks. Rats with urinary protein levels exceeding 30 mg in 24 hours were classified as having T2DN (Supplementary Figure S1B). A total of 80 model rats were prepared, of which four died during the model preparation phase at 4 weeks prior to model establishment (designated as Day 0, Ctrl n=12). On day 0 of the intervention, a total of 35 rats meeting both diagnostic criteria (GLU ≥ 16.7 mmol/L and 24-hour PRO > 30 mg) were selected as

established T2DN models (Supplementary Figure S2).

Subsequently, thirty of these T2DN rats were randomly selected and divided into 3 groups (Supplementary Figure S2, n = 10 per group): the DN model group (DN), which received no cell therapy; the low-dose umbilical cord mesenchymal stem cell group (UMSCs-LD), administered 2×10^6 cells per rat; and the high-dose group (UMSCs-HD), administered 5×10^6 cells per rat. Cell suspensions were delivered via intravenous injection once every two weeks, for a total of three injections. Meanwhile, both the DN model group and a normal control group of SD rats (Ctrl, n = 9) received equal volumes of phosphate-buffered saline (PBS) via the same route and schedule. The timelines for model induction and treatment administration are presented in Figure 1A.

Animal anaesthesia and euthanasia

All rats were euthanized at the designated experimental endpoints. Euthanasia was performed by inducing deep anesthesia via inhalation of 3% isoflurane (in 100% oxygen) in an induction chamber, followed by maintenance at this concentration until the cessation of breathing and cardiac function was verified. All procedures were performed in strict accordance with the animal welfare guidelines approved by the Chinese PLA General Hospital Ethics Committee. Anesthesia was not utilized at any other stage of the study.

Biochemical test

In this study, serial biological samples were collected from rats to monitor physiological and metabolic parameters over a 20-week period. The 24-hour urine samples were obtained at 4-week intervals throughout the 20-week experimental period. Immediately after collection, each sample was centrifuged. This process was conducted at a speed of 4,000 revolutions per minute for a duration of 20 minutes, while the temperature was maintained at 4°C to preserve sample integrity. The resulting supernatant was then carefully separated and analyzed for critical renal and metabolic markers, including urea nitrogen (BUN), 24-hour urinary protein (PRO), and creatinine (UCr). All these analyses were performed in strict compliance with the manufacturer's instructions (XR220PLUS, XinRui, China).

Concurrently, blood samples were drawn from the rats on the same 4-week schedule, culminating at week 20. These samples were subsequently analyzed for a comprehensive panel of metabolic indicators. The assessed parameters encompassed blood glucose, insulin (INS), glycated hemoglobin (HbA1c), triglycerides (TG), and total cholesterol (CHOL). These measurements were carried out using the same Fully Automatic Biochemical Analyzer (XR220PLUS, XinRui, China), meticulously following the standardized protocols supplied by the manufacturer. This synchronized and methodical approach to sample collection and analysis ensured the generation of consistent and reliable longitudinal data for both urinary and hematological biomarkers. The primary outcome measures were blood glucose, HbA1c, and 24-h urine protein.

Pathological staining

Renal tissue samples were fixed in 4% paraformaldehyde and embedded in paraffin according to standard histological protocols. Subsequently, pathological sections of 5 μm thickness were prepared. These sections were subjected to a graded ethanol series for hydration: they were sequentially immersed in 100%, 95%, 70%, and 30% ethanol solutions, each for 2 minutes, followed by a 2-minute immersion in distilled water. For histological evaluation, hematoxylin and eosin (HE) staining was employed to examine the basic

architecture of the glomeruli. Periodic acid–Schiff (PAS) staining was used to assess alterations in the glomerular mesangium and basement membranes, while Masson's trichrome staining was applied to evaluate the extent of renal interstitial fibrosis. All stained sections were reviewed by a pathologist blinded to the experimental conditions. Three random fields of view were selected from each slide, and quantitative analysis was performed using ImageJ software for objective assessment.

Immunohistochemistry/Immunofluorescence

Kidney tissue sections were deparaffinized, rehydrated through graded ethanol and washed with PBS. Antigen retrieval was performed using 10 mM sodium citrate buffer for 20 min. Slides were fixed with 5% BSA for 1 hour at room temperature and then washed with PBS. For ACSL4 (22401-1-AP, Proteintech, China), GPX4 (67763-1-Ig, Proteintech, China), SLC7A11 (26864-1-AP, Proteintech, China), FTH1 (DF6278, Affinity, China), TFRC (AF8136, Beyotime, China), p-ERK (4370S, Cell Signaling Technology, USA) or ANA(ab191181, Abcam, USA) primary antibodies were diluted to the appropriate concentration with 3% BSA, added to the slides and incubated overnight at 4°C environment. After washing, secondary antibodies (ANR02-1, NeoBioscience, China; SA00001-1, Proteintech, China) were added and incubated for 1 hour at room temperature. DBA was then added for a 2-minute reaction, and the sections were reprobed with haematoxylin. The staining intensity was semiquantitatively analyzed using ImageJ software by evaluating at least three random 20× fields per section, which were selected by an independent pathologist (n=5). For ANA detection, the slides were incubated with a red fluorescent-conjugated secondary antibody for 1 hour at room temperature. After washing, the nuclei are counterstained with DAPI for 10 minutes at room temperature. Fluorescence signals are then visualized and captured using a fluorescence microscope equipped with appropriate filter sets.

Cell culture and intervention

The human renal tubular epithelial cell line HK-2 was obtained from China Center for Type Culture Collection (CCTCC No. GDC0152, Wuhan, China) and cultured in MEM (C11095500BT, Gibco, USA) for serial subcultivation. The cells were incubated in a humidified environment at 37 °C with 5% CO2 in MEM medium (C11095500BT, Gibco, USA), supplemented with 10% fetal bovine serum (Gibco, USA), 100 μ/mL penicillin, and 0.1 mg/mL streptomycin (Gibco).

To investigate the effects of different culture conditions, cells were assigned to four experimental groups: (1) Normal glucose control group (NG): cells cultured in 5 mM glucose for 72 hours; (2) High-glucose group (HG): cells exposed to 30 mM glucose for 72 hours to model hyperglycemia; (3) Intervention group (HG+CM): cells treated with 30 mM glucose plus UMSC-conditioned medium (UMSC-CM) for 72 hours; (4) Osmotic control group (Man): cells cultured in 5 mM glucose plus 24.5 mM mannitol for 72 hours to control for osmotic pressure.

Additionally, to inhibit p-ERK activity, cells with HG medium were treated with 10nM GSK2606414 (HY-18072, MCE, China) for 72 h. To determine whether UMSCs trigger p-ERK/ERK pathway to inhibit ferroptosis, HG-cells were treated with UMSCs-CM and 10 μM MK-28 (HY-137207, MCE, China) for 72 h.

ELISA detection of 8-OHdG

The levels of 8-OHdG in kidney tissues or HK-2 cells were determined by ELISA kits

(MM-0331H1, Meimian Jiangsu, China) following the manufacturer's instructions.

Malondialdehyde (MDA) assay

In accordance with the instructions provided in the MDA Assay Kit (S0131S, Beyotime, China), the kidney tissues or HK-2 cells were thoroughly lysed, and the resultant cytoplasm was extracted and combined with the MDA Assay Working Solution, and mixed, and heated at 100°C for 15 min. The resultant mixture was then allowed to cool to room temperature in a water bath, after which it was subjected to centrifugation at 1000 g for 10 min at room temperature. The absorbance at 532 nm was determined by taking the supernatant.

Glutathione (GSH) assay

Kidney tissues or HK-2 cells were lysed according to the procedure of Reduced Glutathione Content Assay Kit (BC1175, Solarbio, China), centrifuged at 12000 g for 10 min at 4°C, and the supernatant was mixed with the assay reagent and allowed to stand at room temperature for 2 min, then the absorbance at 412 nm was measured.

Protein Carbonyl Content (PCO) Test

Kidney tissues or HK-2 cells were collected according to the procedure of Protein Carbonyl Content Assay Kit (BC1275, Solarbio, China) and homogenized by adding the extraction solution, then centrifuged at 4℃, 5000 rpm for 10 min, the supernatant was added with the reagent and left at room temperature for 10 min, then centrifuged at 4℃, 12,000 rpm for 10 min and the supernatant was taken for measurement. Subsequently, the absorbances at 370 nm were measured following the addition of a series of detection solutions.

Prussian Blue Staining (Enhance With DAB)

Kidney tissues sections were deparaffinized to remove paraffin wax and then rehydrated through a graded ethanol series. After rinsing with phosphate-buffered saline (PBS), the tissues were stained with Prussian blue to detect iron deposits. This was followed by a reaction with diaminobenzidine (DAB) for 2 minutes to visualize peroxidase activity. Subsequently, the sections were counterstained with hematoxylin to highlight nuclear structures, mounted with coverslips, and finally observed under a light microscope. For objective quantification, a blinded pathologist randomly selected three non-overlapping fields of view from each stained slide. Three fields of view were randomly selected from each stained slide by a blinded pathologist and quantified using ImageJ (n=5).

Iron array

Total iron levels in tissues were assessed using a tissue iron assay kit (BC4355, Solarbio, China). Rat kidney tissues samples (0.1 g) were harvested and homogenized in 1 mL of extraction solution. All samples were centrifuged at 4000 g for 10 min at 4°C. A total of 120μL of supernatant from each sample was added to the assay reagent with sufficient shaking and centrifuge at 10,000 rpm for 10 min at room temperature. The supernatant was assayed for absorbance at 520 nm immediately.

Western blotting

Western blot was performed as previously described [23]. Briefly, total protein was extracted from either renal tissues or cultured cells using ice-cold RIPA lysis buffer (P0013B, Beyotime, China), supplemented with protease and phosphatase inhibitors. The samples were thoroughly homogenized on ice and subsequently centrifuged at 13,500 × g for 20 minutes at 4°C to pellet cellular debris. The resulting supernatant was carefully collected for further analysis.

Protein concentration was determined using a BCA assay, and equal amounts of protein (10 μg per lane) were denatured in Laemmli sample buffer and subjected to electrophoretic separation on 4%–10% gradient SDS-polyacrylamide gels. The electrophoresis was performed in running buffer at a constant voltage. Following separation, proteins were transferred onto polyvinylidene difluoride (PVDF) membranes using a wet transfer system. The membranes were then blocked with 5% Bovine Serum Albumin (BSA; 36101ES76, Yeasen, China) in Tris-buffered saline containing 0.1% Tween 20 (TBST) for 1 hour at room temperature to prevent nonspecific binding. After blocking, the membranes were incubated overnight at 4°C with the following primary antibodies diluted in TBST with 5% BSA: anti-PGC1α (ab317540, Abcam, USA), anti-TFAM (22586-1-AP, Proteintech, China), anti-ACSL4 (22401-1-AP, Proteintech, China), anti-GPX4 (67763-1-Ig, Proteintech, China), anti-SLC7A11 (26864-1-AP, Proteintech, China), anti-FTH1 (DF6278, Affinity, China), anti-TFRC (AF8136, Beyotime, China), anti-p-ERK (4370S, Cell Signaling Technology, USA), anti-ERK (4695S, Cell Signaling Technology, USA), anti-p-P38 (28796-1-AP, Proteintech, China), anti-P38 (14064-1-AP, Proteintech, China), and anti-β-actin (66009-1-Ig, Proteintech, China). β-actin was used as a loading control to ensure equal protein loading across samples. Following primary antibody incubation, the membranes were washed three times (10 minutes per wash) with TBST and then incubated with appropriate horseradish peroxidase (HRP)-conjugated secondary antibodies (ANR02-1, NeoBioscience, China; SA00001-1, Proteintech, China) for 1 hour at room temperature. Protein bands were visualized using an enhanced chemiluminescence detection system according to the manufacturer's instructions. Band intensities were quantified using ImageJ software (National Institutes of Health, Bethesda, MD, USA). The relative expression levels of target proteins in treated groups were normalized to β-actin and compared to those in control cells or tissues.

ATP level

Intracellular ATP levels were quantified using a commercial ATP Assay Kit (S0026, Beyotime, China) according to the manufacturer's instructions. In brief, cells or homogenized tissues were lysed with the provided lysis buffer. The resulting lysates were subsequently centrifuged at 12,000 g for 5 minutes at 4°C to precipitate insoluble debris. Following centrifugation, the clear supernatant was carefully collected and transferred to a new tube. For the assay, the supernatant was then mixed with the ATP assay working solution, and the luminescent signal was immediately measured using chemiluminescence. A standard curve generated with known concentrations of ATP was run in parallel to calculate the absolute ATP concentrations in the samples.

Mitochondrial Membrane Potential (MMP) assay

Mitochondrial membrane potential was assessed with the JC-1 probe. Collect the appropriate amount of cells, resuspend them in 0.5 mL of cell culture medium, add 0.5 mL of JC-1 probe(M8650, Solarbio, China) staining working solution (50 μL of 200x JC-1 probe was added to 8 mL of ultrapure water to fully dissolve and mix, and then 2 mL of 5x staining buffer was added), and then mix well, and then incubate at 37°C in the cell culture incubator for 20 min. After incubation, the cells were centrifuged at 600 g for 4 min at 4°C to precipitate the cells. The cells were resuspended by adding 1 mL of 1x JC-1 staining buffer, centrifuged at 600 g for 4 min at 4°C and the supernatant was discarded, and the procedure was repeated once. Cells were resuspended with 200 μL of 1x JC-1 staining buffer and analyzed by flow

cytometry.

MtDNA Copy Number

Genomic DNA was isolated from either murine renal cortical tissues or total cultured cells using the commercial DNA Extraction Kit (DP304, TanGen, Beijing, China), strictly according to the manufacturer's protocol. Briefly, approximately 20 mg of snap-frozen kidney tissues or 1×10^6 cells were subjected to lysis, followed by proteinase K digestion. The lysate was then processed through a series of wash steps, and the high-molecular-weight DNA was ultimately eluted in the provided elution buffer. DNA concentration and purity were verified spectrophotometrically, with A260/A280 ratios maintained between 1.8–2.0. Quantitative real-time PCR (qPCR) was performed using an Applied Biosystems QuantStudio 3 Real-Time PCR system. Each 20 μL reaction contained 10 ng of DNA, SYBR Green Master Mix (11201ES03, Yeasen, China), and 0.2 μM of each primer. The thermal profile consisted of initial denaturation at 95°C for 10 min, followed by 40 cycles of 95°C for 15 s and 60°C for 1 min. Melt curve analysis confirmed amplification specificity, and cycle threshold (Ct) values were determined by the instrument software. The primer sequences were as follows:

**Primer name**	**Sequence**
H_mt-ND1-F	CTTCATAGCCGAATACAC
H_mt-ND1-R	GGGGGTTTAAGCTCCTAT
H_mtF3212	CACCCAAGAACAGGGTTT
H_mtR3319	TGGCCATGGGTATGTTGTTAA
H_CYGB-F	CACAAGGTGGAACCGGTGTA
H_CYGB-R	TGGAGTTAGGGGTCCTACGG
R_mt-ND1-F	CACCCCCTTATCAACCTCAA
R_mt-ND1-R	ATTTGTTTCTGCGAGGGTTG
R_CYTB-F	CGGCTGACTAATCCGATACC
R_CYTB-R	TGGGAGTACATAGCCCATGA
R_CYGB-F	GGTGGAACCTATGTACTTTA
R_CYGB-R	GGAAGTCATTGGCAAACT

H: human **R:** rat **CYGB:** cytoglobin **mt-ND1:** mitochondrially encoded NADH dehydrogenase 1 **CYTB:** mitochondrially encoded cytochrome b

ROS level

HK-2 cells were loaded with DCFH probe (S0033S, Beyotime, China) in situ according to the instructions and then incubated at 37°C for 15 min for staining, and photographed by laser confocal microscopy under microscopic observation or assessed via flow cytometry.

RNA-seq and analysis

Total RNA was isolated from rat kidney tissues using Trizol reagent (Invitrogen, USA) followed by RNeasy Mini Kit (Qiagen,USA) purification. rRNA-depleted RNA libraries were prepared with the NEBNext Directional RNA Library Prep Kit. First-strand cDNA was synthesized with random hexamers and M-MuLV reverse transcriptase, followed by second-strand synthesis using DNA Polymerase I and RNase H. Libraries were purified (AMPure XP beads), quality-checked (Agilent Bioanalyzer 2100), and clustered (cBot System, Illumina NovaSeq reagents). Paired-end sequencing was performed on an Illumina NovaSeq platform (Cnkingbio, China). Gene expression was quantified as FPKM. Differentially expressed genes were identified using DESeq2 (v1.30.0) with FDR correction

(Benjamini-Hochberg method).

Statistical analysis

All results were analyzed using GraphPad Prism software (version 10.1.2) and expressed as means ± standard deviation (Mean ± SD). Statistical analysis was performed as indicated in the figure legends. For two group comparison, Student's t-test was performed. For multiple-group comparison, one-way ANOVA analysis was performed.

Supplementary Results

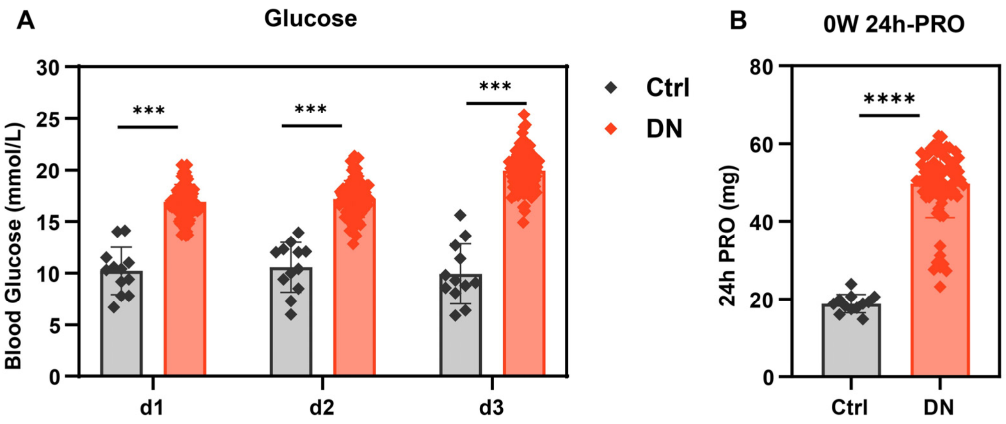

Figure S1 Establishment of the T2DN rat model

A. Blood glucose levels during model induction (Ctrl: n=12, DN: n=80);

B. 24-Hour Urinary Protein Levels at the End of Model Induction (0W n=12/76); *$*p < 0.05$, $**p < 0.01$, $***p < 0.001$.*

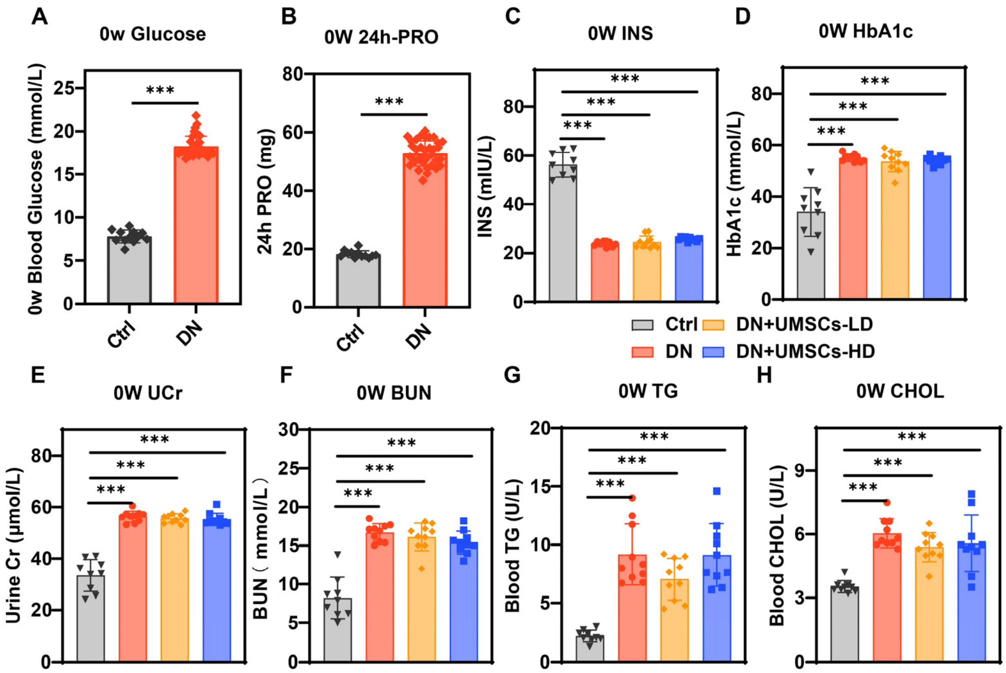

Figure S2 Biochemical parameters upon T2DN model establishment

A-B. Blood glucose and 24h-urinary protein (PRO) levels in rats (Ctrl: n=12, DN: n=35);

C-H. Insulin (INS), HbA1c, creatinine (UCr), urea nitrogen (BUN), triglycerides (TG), and total cholesterol (CHOL) levels in rats following experimental grouping;

(Ctrl: n=9, DN/DN+UMSCs-LD/DN+UMSCs-HD: n=10); *$p < 0.05$, **$p < 0.01$, ***$p < 0.001$.

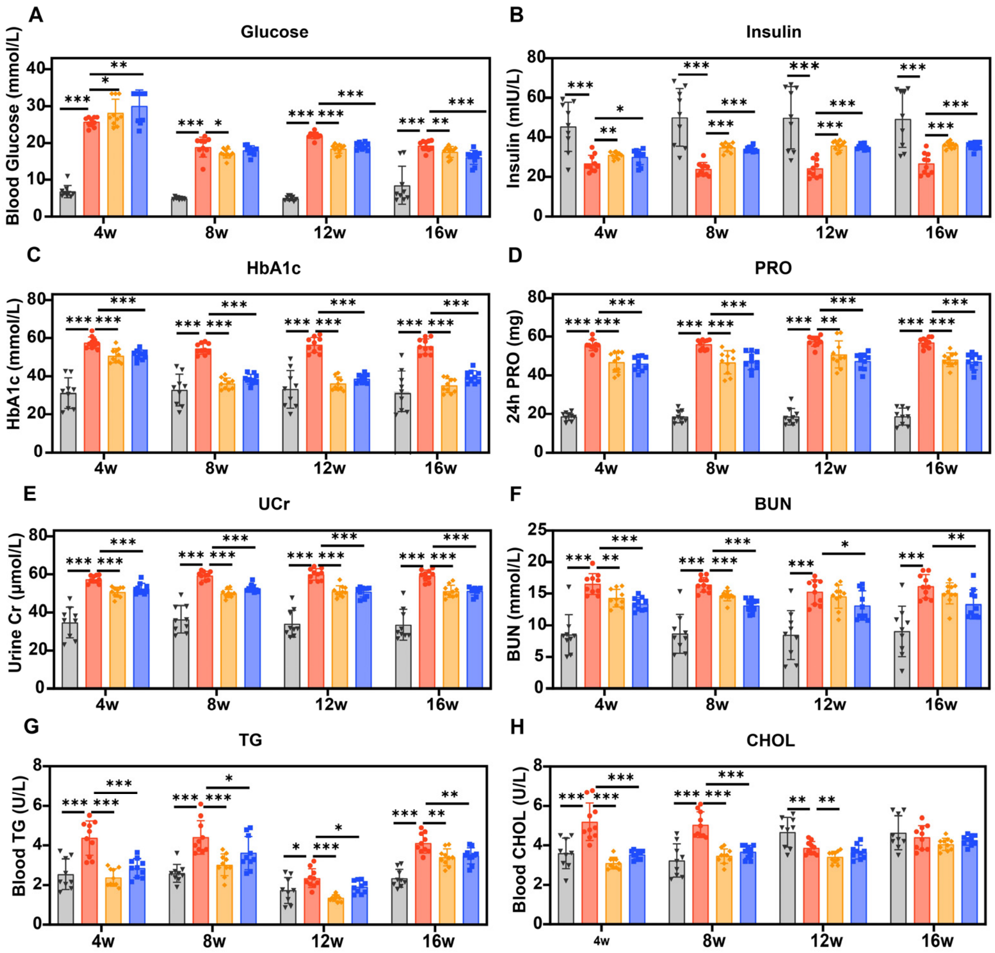

Figure S3 Longitudinal assessment at 4-week intervals during treatment

A-C. Blood glucose, insulin, and HbA1c levels in rats during treatment;

D-F. 24h-urinary protein, urine creatinine (Cr) levels, and urea nitrogen (BUN) levels in rats during treatment;

G. Triglycerides (TG) levels in rats during treatment;

H. Total cholesterol (CHOL) levels in rats during treatment;

(Ctrl: n=9, DN/DN+UMSCs-LD/DN+UMSCs-HD: n=10); *$p < 0.05$, **$p < 0.01$, ***$p < 0.001$.

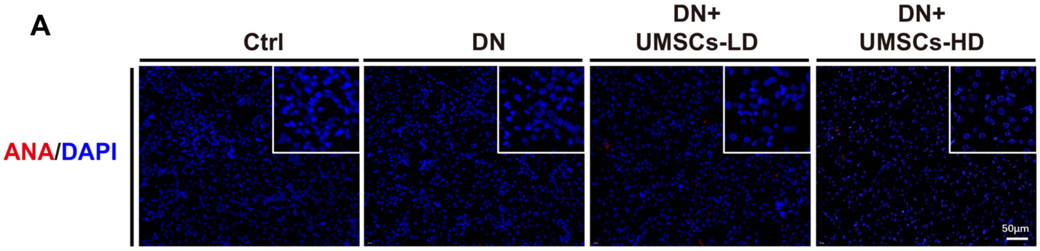

Figure S4 Retention of human MSCs in the DN treatment group at 20 weeks

A. Immunofluorescence analysis of human MSCs retention in the DN group at 20 weeks using anti-human nuclear antibody (ANA, Red). Nuclei were stained with DAPI (blue), n=3, Scale bar=50μm.

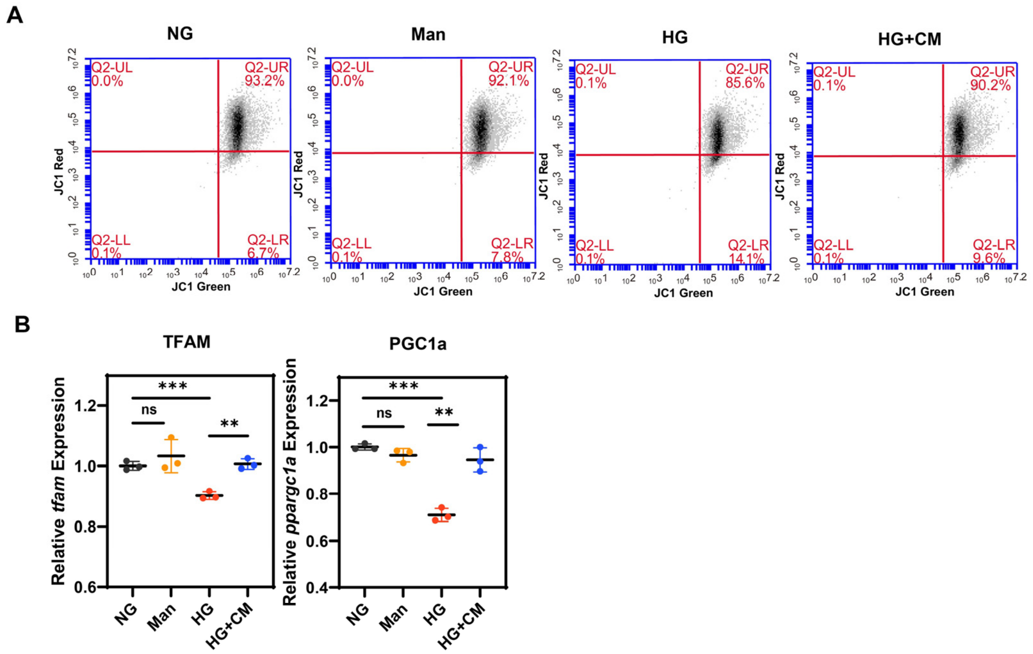

Figure S5 UMSCs-CM reduces high glucose-induced mitochondrial dysfunction in HK-2 cells

A. Mitochondrial membrane potential in Hk-2 cells detected by flow cytometry using JC-1 probe, n=3;

B. Quantitative analysis of mRNA expression levels of *tfam* and *ppargc1a* in HK-2 cells as determined by real-time PCR, n=3; *p < 0.05, **p < 0.01, ***p < 0.001.

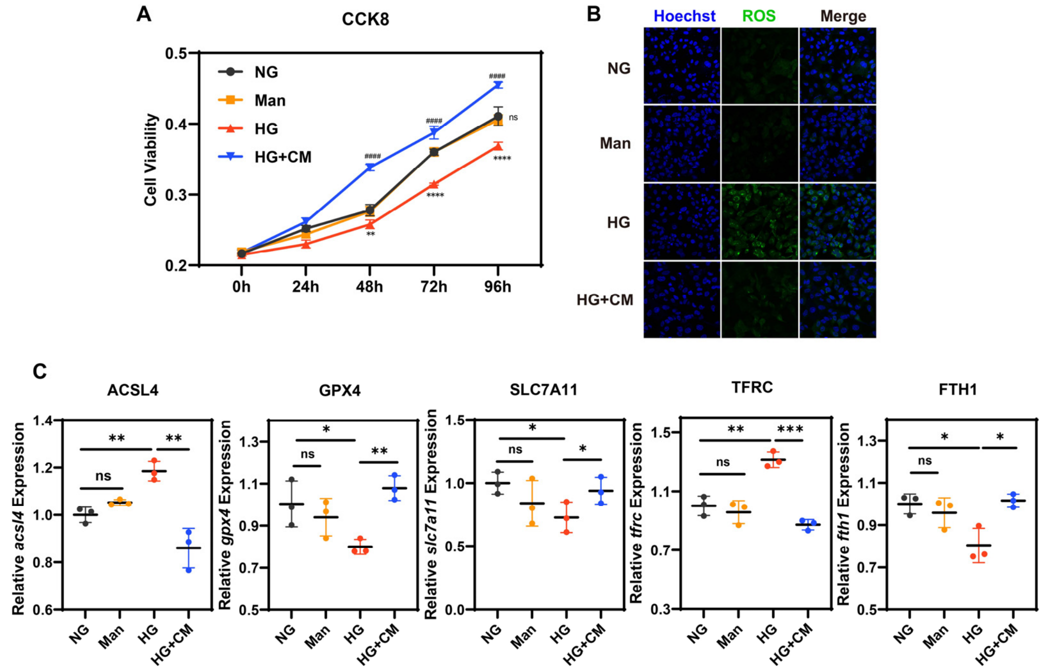

Figure S6 UMSCs-CM reduces high glucose-induced oxidative stress and ferroptosis in HK-2 cells

A. Quantitation of HK-2 proliferation evaluated by CCK-8 kits with the OD value on 0h, 24h, 48h, 72h, and 96h, n=3;
B. Confocal fluorescence imaging of the intracellular ROS scavenging effects of UMSCs-CM using DCFH-DA (green) as the probe. Nuclei were stained with Hoechst 33242 (blue), n=3, Scale bar=50μm;
C. Quantitative analysis of mRNA expression levels of ferroptosis related genes *acsl4*, *gpx4*, *slc7a11*, *tfrc*, and *fth1* in HK-2 cells as determined by real-time PCR, n=3; $^{*}p < 0.05$, $^{**}p < 0.01$, $^{***}p < 0.001$.

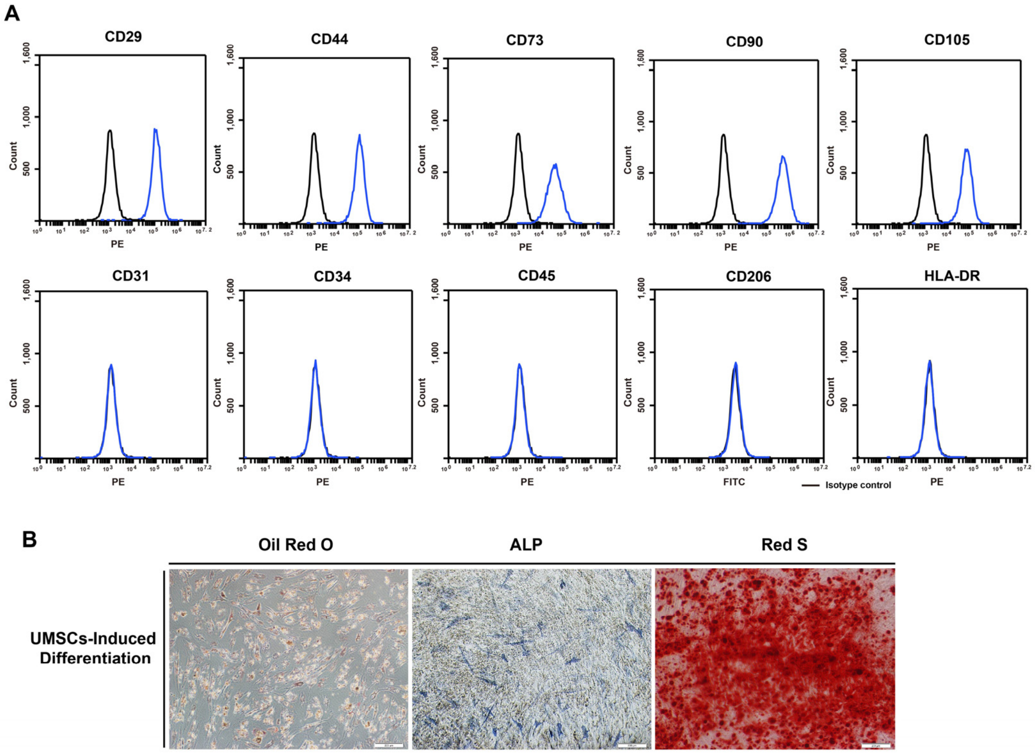

Figure S7 Identification of UMSCs
A. UMSC phenotype analysis by flow cytometry;
B. Representative images of adipogenic and osteogenic differentiation, Scale bar=200μm.
